# Spatiotemporal dipole source localization of face processing ERPs in adolescents: a preliminary study

**DOI:** 10.1186/1744-9081-5-16

**Published:** 2009-03-12

**Authors:** Teresa Ka Wai Wong, Peter Chin Wan Fung, Grainne Mary McAlonan, Siew Eng Chua

**Affiliations:** 1 Department of Psychiatry, The University of Hong Kong, Pokfulam, Hong Kong; 2 Department of Medicine, The University of Hong Kong, Pokfulam, Hong Kong

## Abstract

**Background:**

Despite extensive investigation of the neural systems for face perception and emotion recognition in adults and young children in the past, the precise temporal activation of brain sources specific to the processing of emotional facial expressions in older children and adolescents is not well known. This preliminary study aims to trace the spatiotemporal dynamics of facial emotion processing during adolescence and provide a basis for future developmental studies and comparisons with patient populations that have social-emotional deficits such as autism.

**Methods:**

We presented pictures showing happy, angry, fearful, or neutral facial expressions to healthy adolescents (aged 10–16 years) and recorded 128-channel event-related potentials (ERPs) while they performed an emotion discrimination task. ERP components were analyzed for effects of age and emotion on amplitude and latency. The underlying cortical sources of scalp ERP activity were modeled as multiple equivalent current dipoles using Brain Electrical Source Analysis (BESA).

**Results:**

Initial global/holistic processing of faces (P1) took place in the visual association cortex (lingual gyrus) around 120 ms post-stimulus. Next, structural encoding of facial features (N170) occurred between 160–200 ms in the inferior temporal/fusiform region, and perhaps early emotion processing (Vertex Positive Potential or VPP) in the amygdala and orbitofrontal cortex. Finally, cognitive analysis of facial expressions (P2) in the prefrontal cortex and emotional reactions in somatosensory areas were observed from about 230 ms onwards. The temporal sequence of cortical source activation in response to facial emotion processing was occipital, prefrontal, fusiform, parietal for young adolescents and occipital, limbic, inferior temporal, and prefrontal for older adolescents.

**Conclusion:**

This is a first report of high-density ERP dipole source analysis in healthy adolescents which traces the sequence of neural activity within the first 500 ms of categorizing emotion from faces. Our spatio-temporal brain source models showed the presence of adult-like cortical networks for face processing in adolescents, whose functional specificity to different emotions appear to be not yet fully mature. Age-related differences in brain activation patterns illustrate the continued development and maturation of distinct neural systems for processing facial expressions during adolescence and possible changes in emotion perception, experience, and reaction with age.

## Background

Discerning facial emotions is fundamental for normal social interaction and commences in early infancy [1]. Recognition and interpretation of emotion from facial expressions occurs almost instantaneously and is used in social referencing, for example, when an infant first encounters a novel object and looks towards the parent to direct their behavior. If the parent expresses a happy face, the child will understand this to be an encouragement to approach the object [2]. On the other hand, if the parent displays an expression of fear or disgust, the child will tend to avoid the same object. This skill of "reading" emotions from faces becomes more proficient as children grow up and experience a multitude of social, emotional and cognitive changes. However, the neural circuitry that controls facial emotion processing during late childhood and adolescence has received scant attention to date [3]. This period of cognitive and behavioral development is critical since heterochronous brain changes take place: cortical synaptic elimination occurs in the primary visual and auditory cortices during late childhood, then subsequently in the prefrontal cortex in mid-adolescence [3]. Compatible with this is the region-specific nonlinear increase in cortical grey matter volume, which peaks in the frontal and parietal lobes around age 12, followed by the temporal and occipital lobes at about ages 16 and 20 respectively [4].

Neurobiological findings from lesion, functional magnetic resonance imaging (fMRI) and positron emission tomography (PET) studies have revealed that, just as distinct brain structures subserve verbal language, specialized neural networks also exist for processing the non-verbal language of facial expressions. These include the occipito-temporal cortices, amygdala, orbitofrontal cortex, as well as somatosensory-related cortices in the right hemisphere [5]. Studies of pervasive developmental disorders such as autism have also identified abnormalities in the fusiform gyrus, superior temporal sulcus, and amygdale [6], suggesting that the innate inability to perceive and respond to complex emotional expressions of others may parallel early abnormal development of brain regions involved in face processing. Prosopagnosic patients who demonstrate impairments in recognizing familiar faces, but have normal abilities in identifying facial expressions of emotion [7], suggest a functional dissociation between facial identity and facial expression processing [8]. In accordance with Bruce and Young's cognitive model for face perception [8], Haxby, Hoffman, and Gobbini's neuroanatomical model [9] posits that, after the initial stage of face perception has taken place in the inferior occipital gyri, (bidirectional) information proceeds along two distinct parallel neural pathways: one for coding facial identity (lateral fusiform gyrus for recognizing unique invariant facial features), and the other for coding facial expression (superior temporal sulcus region for analyzing the changeable aspects of faces e.g. eye gaze and lip movement). The models of Bruce and Young [8] and Haxby et al. [9] however, do not include the precise time frame in which the various components of face processing take place.

Such time-sensitive issues can be addressed using dense-array electroencephalography (EEG), event-related potentials (ERPs), and magnetoencephalography (MEG), since these techniques provide millisecond temporal resolution not accessible by hemodynamic measures. Recent work has localized sources of face-specific components to the occipital and inferotemporal cortices [10,11], which overlap with regions identified in fMRI studies [12-14]. For example, an equivalent current dipole model for adult face recognition showed the following temporal dynamics of ERP source activation: bilateral lingual gyri at 120 ms, followed by the right and then left fusiform/hippocampal gyri around 150–170 ms, bilateral medial temporal gyri from 200 ms onwards, the right caudate nucleus peaking at 300 ms, and finally the anterior cingulate gyrus 300 ms after stimulus onset. However, such source localization has not yet been performed on ERP data from children and adolescents actively processing facial expressions of emotion, and only few studies to date have examined differential neural processing of emotional facial expressions in children [15,16] and adolescents [17], let alone using high-density EEG.

Adult studies have suggested that emotionally expressive faces (especially high-arousal negative emotions like fear or anger) tend to evoke larger ERP responses than emotionally unexpressive (neutral) faces [15,16,18]. The "face-sensitive" N170 component over posterior temporal scalp regions is said to reflect the encoding of configural and relational features within a face [19] and appears not to be selective for any particular facial expression [17,20-23]. However, others have found that negative (fear and disgust) emotions do indeed evoke larger N170 amplitudes in adults than positive (happy, surprise, neutral) ones [24,25]. Similarly, the vertex positive potential (VPP), originating from the same pair of dipoles in the lateral inferior occipital cortex/posterior fusiform gyrus that generates the N170 in adults [26], is enhanced by facial expressions of fear [20,21]. In addition, the timing of the neural response in adults seems also affected by emotion, such that the N170 appears at earlier latencies for positive than negative emotions. Krolak-Salmon et al. [23] reported that occipital ERP patterns first distinguished between emotional and neutral faces during the 250–550 ms time interval, and then differed between emotional expressions (fear and happiness in particular) from 550–750 ms over right occipito-temporal scalp regions. Activity specific to expressions of disgust occurred even later, between 700–900 ms in frontal and right temporal areas [23].

Several studies of young children (3–7 years of age) have shown differential ERP activity to facial emotion at latencies longer than about 300 ms [27-29], but earlier emotion effects on the face-elicited P1, N170, or P2 components as seen in adults were not present in children between 9–15 years [17]. In contrast, Batty and Taylor [30] detected an effect of emotion on the P1 latency in children as young as four years old. This early emotion effect, indexing rapid holistic processing of faces [31], however, disappeared with age (absent after 7 years old) along with age-related decreases in P1 amplitude and latency. By mid-teens, global processing of emotions was replaced by detailed configural processing, reflected by an emotion effect on the N170 amplitude in 14- to 15-year-olds [19,30]. This change in the spatio-temporal profile of face-processing ERPs with development [32] suggests that as cortical circuits mature and become more specialized for processing faces, dipole generators of ERP components may shift in location or orientation from childhood to adolescence [33]. For example, it is thought that two functionally distinct cortical generators of the N170a and N170b subcomponents in children are fused into a single source responsible for the adult N170 [32]. One model of adult face encoding and recognition [19] explained the posterior P1 and P2 components with bilateral dipole generators in the parietooccipital cortex, the N170 component by a pair of posterior ventral dipoles, and the VPP component by an orbitofrontal regional dipole source in the right hemisphere. However, no study has yet investigated the dipole source location and time course of processing facial expressions of emotion in the adolescent years.

The main purpose of this study was to construct a dipole model of brain sources for healthy adolescents to show the temporal unfolding of neural events that occur during explicit processing of different facial emotions. In addition, we examined the effects of emotion on ERP amplitudes, latencies, and scalp distribution, as well as developmental changes in ERPs to faces from early (age 10 to 13 years) to late (age 14 to 16 years) adolescence. We hypothesized that facial-emotional processing would occur rapidly in time, sequentially activating brain regions similar to those seen in adults subserving face detection, structural encoding of facial features, and emotion recognition.

## Methods

### Participants

Twenty healthy right-handed Chinese adolescents (12 male, 8 female) aged between 10 and 16 years (mean 14.2 ± 1.8 years) took part in this study. In order to examine developmental changes in face-specific ERPs and their sources [34], participants were divided into two groups: 10 young adolescents aged 10–13 years, and 10 older adolescents aged 14–16 years. However, due to severe artifact contamination in four young participants, we analysed 6 datasets (3 male) in the young group and 10 datasets (5 male) in the old group.

The research protocol was approved by the Institutional Review Board of the hospital where the study took place and all participants were compensated for travel and inconvenience. Parents of participating children signed informed consent and every participant gave their assent to participate in the project. All were screened for psychiatric illness using the parental Chinese Diagnostic Interview Schedule for Children for DSMIV [35] and had no history of headache, loss of consciousness, nor any neurological disorder. All attended normal school and had normal or corrected-to-normal vision.

### Stimuli and procedure

**Figure 1:**
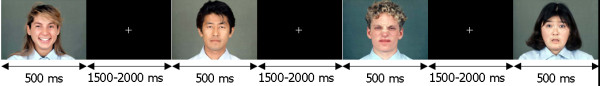
**Stimulus sequence**. Schematic diagram of a typical trial sequence of face stimuli used in explicit and implicit emotion processing tasks, with timing parameters as shown.

ERP stimuli consisted of 32 color photographs selected from the standardized set of Japanese and Caucasian Facial Expressions of Emotion (JACFEE) and Neutral Faces (JACNeuF) [36]. The images of four males and four females each depicting happy, angry, fearful, and neutral facial expressions were randomized six times to generate a sequence of 192 stimulus trials. Participants were instructed to press one of four buttons corresponding to 'happy', 'angry', 'fearful', or 'neutral' expressions, in response to the face stimuli presented. As shown in Figure 1, each face was presented for 500 ms on the full screen of an 11- by 13-inch monitor situated about one meter in front of the participant. A central fixation '+' was displayed during inter-stimulus intervals of lengths varying between 1500–2000 ms. E-Prime v.1.0 beta (Psychology Software Tools, Inc.) was used to present the visual stimuli and to record reaction times and button-press responses. During EEG recording, the attentive state of the participant was monitored and short breaks were permitted after each 2-minute block of 48 trials to allow the participant to rest.

### EEG recording and signal processing

Scalp EEG was recorded using a 128-channel Geodesic Sensor Net (Electrical Geodesics, Inc., Eugene, OR, USA) that matched the head size of the participant. Electrode impedances were adjusted to stabilize under 50 kΩ before recording, and continuous EEG was sampled at 500 Hz with filters 0.1 to 100 Hz using the vertex (electrode 129) as reference. Offline, notch filters were employed to remove electrical noise from the mains power supply (50 Hz) and the stimulus display monitor (60 Hz refresh rate). Subsequent data processing was done using Brain Electrical Source Analysis (BESA 5.1, MEGIS Software GmbH, Munich, Germany). Eye-blink patterns were marked and averaged for each individual and the spatial topography of the blink artifact was used for correction of individual EEG data by the surrogate model approach [37]. The surrogate model for brain activity consisted of 15 regional sources covering all brain regions. After blink correction, trials with remaining artifacts between -100 ms and 500 ms (amplitude > 100 uV, gradient > 75) were rejected and a 1 Hz high-pass forward filter was applied to reduce slow channel drift as well as to provide implicit baseline correction. EEG epochs, starting 200 ms prior to stimulus onset until 800 ms post-stimulus, were averaged for each emotion category. The resulting individual ERPs were transformed to an average reference and low-pass filtered at 30 Hz for ERP analysis and dipole source modeling.

### ERP analysis

A mean of 37 ± 9 (SD) clean trials were averaged for each emotion per participant. ERPscore [38] was used to extract the individual peak amplitude and latency values for each ERP component in the four emotion conditions. Time windows for scoring ERP peaks were determined from plots of global field power of individual participant averages, and amplitudes were measured from electrodes where the component was largest. The posterior component P1 was scored at occipital sites 76 (Oz), 72, and 77, and the anterior N1, VPP, and N2 components were measured from fronto-central sites 6 (FCz), 7, and 107. The N170 component was measured from bilateral temporal sites 57, 58 (T5), 64, and 96, 97 (T6), 101, and the P2 from bilateral parietal sites 59, 60 (P3), 66, and 85, 86 (P4), 92.

**Figure 2:**
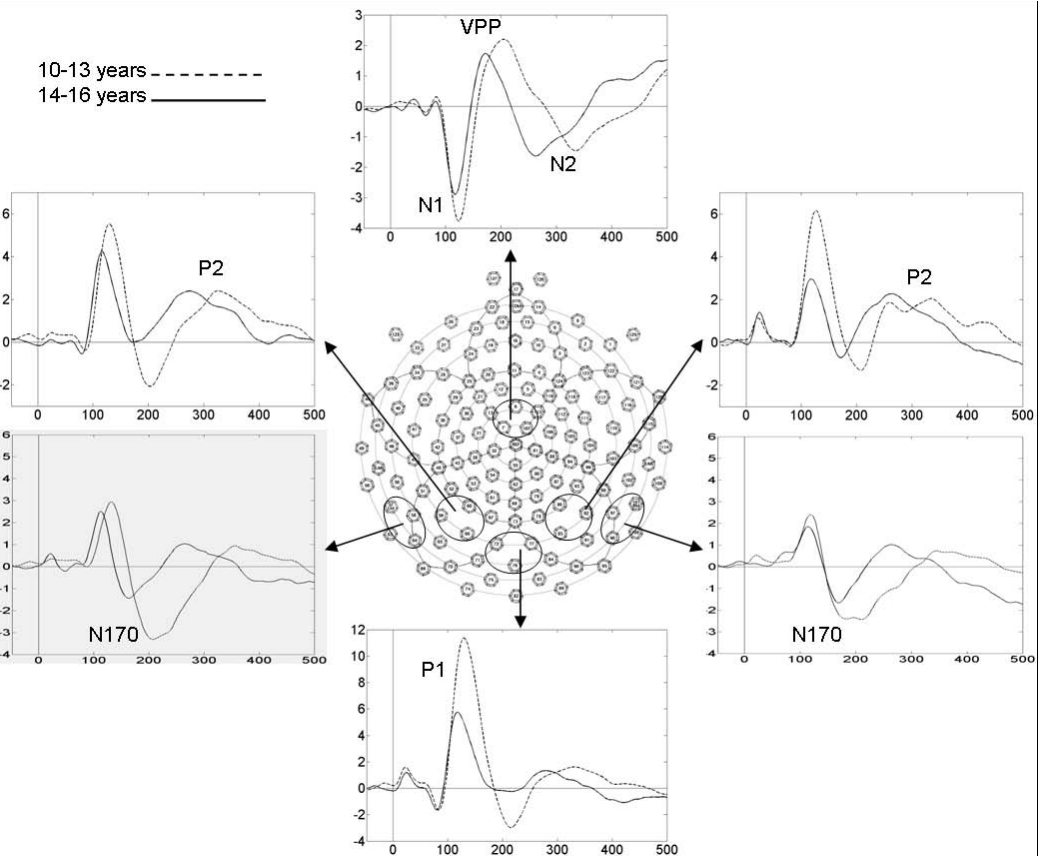
**Scalp ERPs to faces**. Combined-conditions grand-average ERP waveforms for 10- to 13-year-olds (dashed lines) and 14- to 16-year-olds (solid lines) collapsed across selected electrode groups. Vertical scale represents voltage amplitude in uV and horizontal scale displays latency in ms.

Our selected scalp regions for ERP analysis (see Figure 2) correspond to electrode sites commonly used in similar high-density ERP studies [17,18,39]. Instead of peak amplitude, average amplitude was used to measure the slower P2 and N2 components across seven 30-ms time windows between 220 and 430 ms [30]. Repeated measures analyses of variance (ANOVA, using SPSS 14.0 for Windows) on ERP amplitudes and latencies were performed using Emotion (happy, angry, fearful, neutral), Hemisphere (left, right for N170 and P2 components), and Electrode (3) as within-subject factors and Age Group (10–13 years, 14–16 years) as the between-subject factor. Greenhouse-Geisser corrected degrees of freedom were used whenever the sphericity assumption was violated [40].

### Dipole source analysis

Brain Electrical Source Analysis (BESA v.5.1, MEGIS Software GmbH, Munich, Germany) was used to model cortical sources of ERPs as equivalent spatiotemporal current dipoles with a certain orientation and time-varying dipole moments [41]. Separate source models were created for the 10–13-year-olds and the 14–16-year-olds in realistic isotropic head models with conductivity ratio of 60 for the young adolescents and 80 for the older group [42-44]. Visual inspection of scalp topographies at latencies of peak ERP activity suggested that placing a symmetrical constraint on the location of bilateral pairs of sources was appropriate in order to limit the number of parameters in the inverse problem. Principal component analysis (PCA) decomposition showed that four pairs of dipole sources would sufficiently explain the majority of the variance in the ERP waveform.

Four pairs of sources were fit sequentially over time at latencies of the P1, N170/VPP, and P2 components, then adjusted in an iterative manner until they localized to stable locations and orientations, with the best temporal separation of source waveforms and as little interaction between sources as possible. The fit interval assigned to each pair of sources was defined such that the decomposition of the residual data (unexplained by previously fitted sources) was dominated by a single PCA component. Since preliminary source analysis on the grand average ERPs to each of the four different facial expressions did not show qualitative differences in source location between emotions, the source model for each age group was constructed based on the combined-conditions (all facial expressions) grand average ERP [45,46]. The final source solutions required a residual variance of less than 10% [37,47], i.e. a goodness of fit over 90%.

## Results

### Behavioral data

Significant main effects of Emotion on both accuracy [F(3,39) = 24.328, p < 0.001] and reaction time [F(3,39) = 39.004, p < 0.001] showed that happy faces were recognized most quickly (mean reaction time 761.5 ms) and accurately (mean 97.0% correct), while angry expressions took the longest to discriminate (mean reaction time 968.1 ms) and were more likely to be misclassified (mean 75.4% correct). The older group were overall more accurate (mean 92% correct) than the younger group (mean 83% correct) in identifying facial expressions [F(1,13) = 6.099, p = 0.028], but their mean reaction times did not differ significantly. A significant Emotion by Age Group interaction [F(3,39) = 7.339, p = 0.001] revealed that younger adolescents misclassified angry expressions more often than any of the other expressions (all p < 0.05) and recognized happy faces more easily than fearful faces (p = 0.024). Older adolescents were equally proficient at identifying happy, fearful, and neutral faces but also found angry expressions most difficult to discriminate (p = 0.018).

### ERP results

Figure 2 shows ERP waveforms averaged over all four expressions and electrodes of interest for the two age groups.

*P1*: The occipital P1 peaked at a mean latency of 122 ms in 10- to 13-year-olds and 114 ms in 14- to 16-year-olds, but effects of Emotion, Electrode, and Age Group on amplitude and latency did not reach statistical significance.

*N1*: The fronto-central N1, with mean latencies of 118 and 115 ms in the younger and older age groups respectively, was maximal over electrode FCz [F(2,28) = 3.568, p = 0.042] but did not show significant effects of Emotion or Age Group.

**Figure 3:**
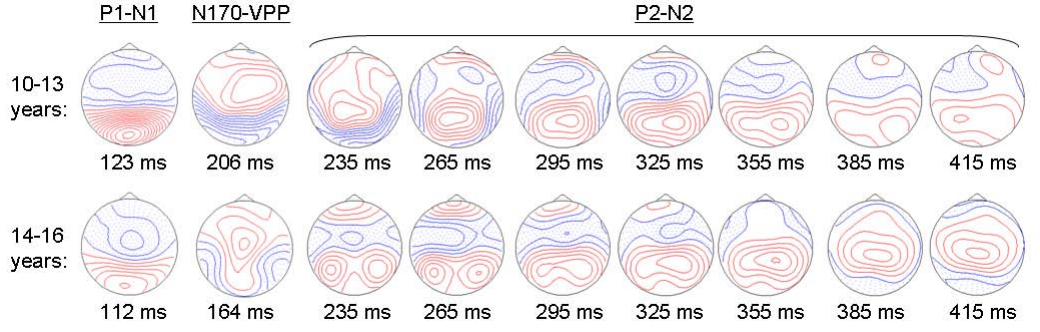
**ERP scalp maps to faces**. Voltage topography maps showing the scalp distribution of P1-N1 and N170-VPP dipolar complexes at latencies of maximum global field power for 10- to 13-year-olds (top row) and 4- to 16-year-olds (bottom row). The P2-N2 complex is shown over the seven 30-ms analysis time windows. Shaded areas show negative voltages and each contour represents a step of 0.5 uV.

*N170*: There were no significant effects of Emotion, Hemisphere, Electrode, or Age Group on the N170 latency, which averaged 189 ms in 10- to 13-year-olds and 173 ms in 14- to 16-year-olds. All effects on N170 amplitude were also insignificant. Although Figure 3 shows a more widespread occipital N170 distribution in 10- to 13-year-olds and a more bilateral temporal distribution in 14- to 16-year-olds, this difference in N170 topography did not reach statistical significance (no significant Electrode by Age Group interaction was found when a larger group of temporal-occipital electrodes were analysed).

*VPP*: The VPP showed a trend of earlier latencies in the older adolescents compared to the younger ones [mean latency 175 ms versus 196 ms respectively, F(1,14) = 4.095, p = 0.063].

*P2*: No significant effects of Emotion, Hemisphere, Electrode, or Age Group were observed for the parietal P2 mean amplitudes across the seven 30-ms time windows between 220 and 430 ms. However, significant Electrode by Age Group interactions were found in time intervals 250–280 ms and 340–430 ms (p < 0.05) of the P2 wave, as illustrated by the age-related differences in P2–N2 topographies shown in Figure 3.

*N2*: The fronto-central N2 was also unaffected by Emotion or Electrode, and Age Group effects were present only in the last two time windows between 370–430 ms, reflecting the delayed N2 peak in young relative to older adolescents (see Figure 2).

### Dipole sources of ERPs

**Figure 4:**
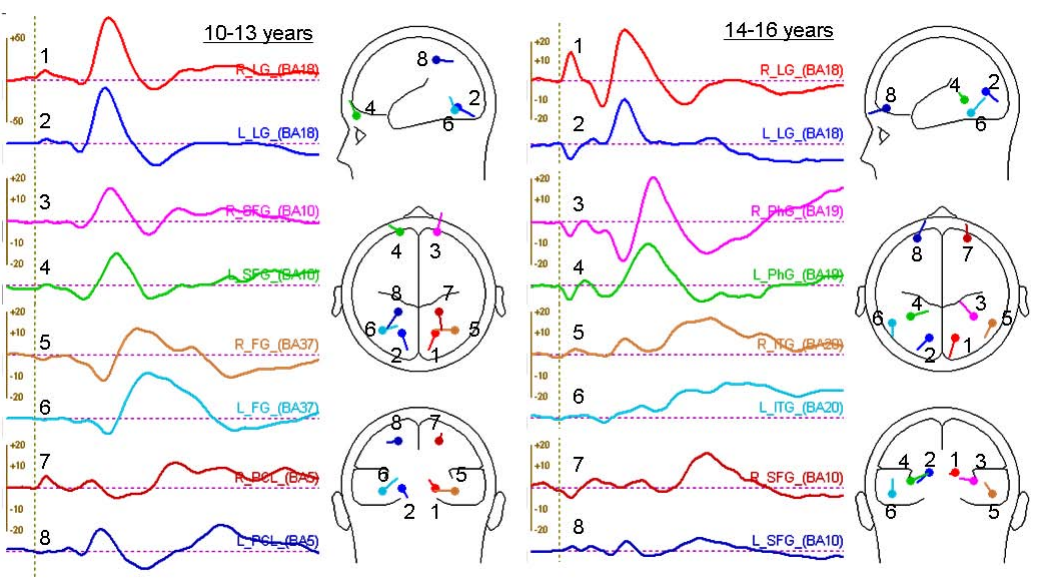
**Dipole source waveforms and cortical locations**. BESA solutions for 10- to 13-year-olds (left panel) and 14- to 16-year-olds (right panel) showing time varying source activity (dipole moments in nAm) and the estimated 3D dipole locations and orientations within the head model.

Figure 4 illustrates the time-varying cortical activity that explains the scalp ERP components and the slightly different dipole locations and orientations within the three-dimensional head model of the two groups. Talairach coordinates of the dipole locations are listed in Tables 1 and 2, together with an estimation of their nearest corresponding anatomical structure and latencies of peak source activation. With the caveat that discrete equivalent current dipoles only represent idealized point sources on an active patch of cortex [48], each source waveform is likely to describe activity of the named structure and/or integrated activity from the 'center of mass' of several neighboring structures within a one centimeter radius. Nevertheless, our two source models explained 93% and 91% of variance in the group averages of 10- to 13-year-olds and 14- to 16-year-olds respectively within the analysis time window of 90 to 400 ms.

**Table 1:** Dipole locations and latencies of maximum dipole moments in source model for 10- to 13-year-olds

Dipole source	Talairach coordinates (mm)			Nearest anatomical structure (Brodmann area)	Dipole moment (nAm)	Latency (ms)
	x	y	z			

1	17.4	-66.5	-9.7	Right lingual gyrus (BA18)	73.4	130
2	-17.4	-66.5	-9.7	Left lingual gyrus (BA18)	65.8	124
3	19.3	67.0	-5.3	Right superior frontal gyrus (BA10)	15.4	132
4	-19.3	67.0	-5.3	Left superior frontal gyrus (BA10)	14.7	142
5	37.5	-62.2	-12.3	Right fusiform gyrus (BA37)	12.2	180
6	-37.5	-62.2	-12.3	Left fusiform gyrus (BA37)	21.1	198
7	20.8	-45.3	46.3	Right paracentral lobule (BA5)	11.7	246
8	-20.8	-45.3	46.3	Left paracentral lobule (BA5)	12.2	324

**Table 2:** Dipole locations and latencies of maximum dipole moments in source model for 14- to 16-year-olds

Dipole source	Talairach coordinates (mm)			Nearest anatomical structure (Brodmann area)	Dipole moment (nAm)	Latency (ms)
	x	y	z			

1	13.6	-74.2	7.6	Right lingual gyrus (BA18)	22.8	114
2	-13.6	-74.2	7.6	Left lingual gyrus (BA18)	22.8	114
3	32.9	-45.8	1.4	Right parahippocampal gyrus (BA19)	20.7	164
4	-32.9	-45.8	1.4	Left parahippocampal gyrus (BA19)	19.2	156
5	52.3	-52.1	-14.0	Right inferior temporal gyrus (BA20)	17.0	266
6	-52.3	-52.1	-14.0	Left inferior temporal gyrus (BA20)	16.1	294
7	26.4	56.9	1.6	Right superior frontal lobule (BA10)	16.2	260
8	-26.4	56.9	1.6	Left superior frontal lobule (BA10)	6.1	244

The posterior P1 scalp component in all participants was characterized by a pair of occipital sources localized in the lingual gyrus bilaterally. Activation of this first pair of sources in the visual cortex was particularly strong in the young adolescents at their P1 peak latency (122 ms), reflecting their larger P1 amplitude relative to the older group, whose dipoles were situated more superiorly. Within this early time frame, the 10- to 13-year-olds also activated sources in the frontopolar region near the superior frontal gyrus symmetrically, which helped to explain their relatively larger fronto-central N1 peak.

Next, a pair of sources in the temporal lobes around the fusiform gyrus accounted for the 10- to 13-year-olds' N170 just before 200 ms, and bilateral sources in the superior parietal lobe contributed to their P2 and N2 components around 300 ms. In contrast, the source model for 14- to 16-year-olds showed limbic activity originating near the parahippocampal gyrus at about 160 ms (N170) after the initial visual (P1) response in the lingual gyrus at 114 ms. Generators of the subsequent P2 and N2 components were estimated in the inferior temporal and prefrontal regions with greater source strengths seen in the right hemisphere than the left.

## Discussion

This study examined the precise temporal dynamics of adolescent neural processing in response to judging emotion from facial expressions and provided a first report of multiple dipole source analysis on healthy adolescent data. In 10- to 13-year-olds, sequential activation of occipital, prefrontal, fusiform, and parietal brain sources were observed, whereas in 14- to 16-year-olds, neural sources in the occipital, limbic, inferior temporal, and finally prefrontal region were successively activated in response to face processing. The similarity in source locations between our adolescent models and previous reports of dipole source analyses on adult data [11,19,49-51] suggest that cortical networks for face-specific processing are present before 16 years of age [52,53], but that their functional specificity to different emotional expressions are not yet fully mature. Differences in brain sources and activation patterns between young and older adolescents detected in this study provide preliminary evidence for the continued development and maturation of distinct neural systems for processing emotional facial expressions during adolescence and possible changes in emotion perception, experience, and reaction with age.

Our behavioral data confirmed the improvement of facial emotion recognition with age [54], and that happiness is universally the most accurately recognized facial emotion [55]. Angry expressions on the other hand, are likely perceived as more complex than other emotions [29] and hence took the longest to discriminate and were misclassified most often by adolescents, while neutral expressions might have been emotionally ambiguous. The lower accuracy rate in 10- to 13-year-olds could also substantiate the temporary performance dip in face encoding/recognition [56,57] and cognitive/emotional processing [58] during this period of synaptic reorganization at the onset of puberty.

Our ERP results support the hypothesis that the P1 component reflects an initial response to visual stimuli and the early global/holistic processing of faces [31], while the N170 represents a subsequent encoding stage of configural and relational features within a face [19]. Consistent with developmental ERP studies of facial expression processing [30,32], we observed a decrease in ERP peak latencies as well as a reduction in the occipital P1 amplitude from the young to old adolescent groups, reflecting increasing cortical efficiency in face processing with age. The N170 voltage topography showed a shift from a widespread occipital negativity in 10- to 13-year-olds to a more focal lateral temporal distribution in 14- to 16-year-olds, providing support for a change in location and/or orientation of its dipole generator(s) as neural circuits mature and become more specialized for processing faces [33]. Continuing maturation of the face-specific N170 through adolescence is compatible with previous work suggesting that between the ages of 10 and 13 years, two functionally distinct cortical generators of the N170a and N170b subcomponents in children begin to fuse into a single source responsible for the adult N170 [32]. Developmental fMRI studies have also supported differences in the engagement and modulation of neural systems involved in face processing between adolescents and adults [59,60]. Our finding that early ERP components in adolescents were insensitive to emotion agrees with a similar study [17] in which emotion effects were present only in adults but not in children. We presume that face-specific ERP components which seem to distinguish between emotional expressions in adults [16,21,24] are still immature in early adolescence, and that emotion effects and hemisphere asymmetry may only become visible when the adult N170 morphology develops fully sometime after the mid-teens [32]. However, due to the limited sample size of this study and our attempt to explore developmental differences in ERPs and their sources during the adolescent period, significant effects of emotion and age may possibly have been masked by large inter-subject variability in brain responses during pubertal development. Further confirmatory studies using larger participant numbers and narrower age bands will be important for more in-depth examination of developmental changes in ERP sources as well as sex-related differences in brain responses to different facial emotions [61].

Using BESA, we constructed two equivalent current dipole models to explain ERP activity in the two adolescent age groups. Both models contained sources in the visual association cortex (lingual gyrus, LG) and around the inferior temporal/fusiform gyrus (FG) that explained the P1/N1 and N170/VPP dipolar complexes respectively. Occipito-temporal sources including the LG and FG have been previously identified in adult studies as dipole generators of face-sensitive ERPs [11,19,49-51] and MEG signals [62,63]. Although the accuracy of source localization is limited to the spatial sampling of scalp signals as well as the statistical and geometrical properties of the brain and head model used [64], our source locations and activation time courses corresponded well with adult intracortical ERP recordings [65,66], MEG sources [62,63,67], and fMRI activations [12,13,68,69] to face stimuli. Halgren et al. [65] located an intracortical N75-P105 response (cf. our P1) to faces in the LG (visual areas 17 and 18), followed by an N130-P180-N240 complex (cf. our scalp N170-VPP-N2) in the FG (Brodmann areas 19 and 37) and the posterior superior and middle temporal gyrus. They also showed a wide transmission of the face-specific potential (P180) from the basal temporo-occipital cortex to the superior temporal sulcal, parietotemporal, and dorsolateral prefrontal cortices [65]. With extensive reciprocal connections between temporal visual regions and the prefrontal cortex, it is postulated that activity in the temporal cortex can modulate input to the orbitofrontal cortex during facial emotion processing [5].

Indeed, adult studies using fMRI and PET have demonstrated significant involvement of frontal brain regions for explicit identification of facial emotion [13,68-70]. Furthermore, adults have been found to produce greater activation in the orbitofrontal cortex than adolescents regardless of whether attention is directed to emotional or nonemotional aspects of a face [59]. According to structural MRI studies, structures in the frontal lobe are still developing post-adolescence [71], as evidenced by significant loss of frontal gray matter due to synaptic pruning between adolescence and adulthood and continued axonal myelination that increases white matter density with age [72]. We believe that structural changes in the prefrontal cortex during adolescent brain development may account for the difference in activation patterns of prefrontal sources observed in our two dipole models. Our adolescent source locations are consistent with a previous model of adult face encoding and recognition [19] that explained the VPP component by an orbitofrontal regional dipole source in the right hemisphere, together with bilateral dipole generators of the posterior P1 and P2 components in the parietooccipital cortex and a pair of posterior ventral sources accounting for the N170. Similarly, Sabbagh et al. [73] localized the frontal N270–400 elicited during mental state decoding from pictures of eyes to the orbitofrontal and medial frontal cortices in adults using low-resolution electromagnetic tomography (LORETA-KEY). They contended that the right orbitofrontal region is used for understanding others' mental states, while the left medial frontal regions are engaged in theory-of-mind reasoning about mental states [73]. Strong right-lateralized activation of the frontopolar region in our 14- to 16-year-old adolescents likely reflects the conscious evaluation and judgment of emotion category [69,70] and is also suggestive of recognition of previously seen faces [74]. Recruitment of more mature frontal networks for social cognition and executive function in older adolescents [72] is expected to facilitate emotional processing and explains their superior performance in discriminating facial expressions compared to the younger group.

In addition to occipital, temporal, and prefrontal sources of ERPs, we also detected parahippocampal activity in the older adolescents around 160 ms. The parahippocampal gyrus has been speculated to be part of a ventro-medial pathway that receives input from the lingual and fusiform gyri and transmits perceptual information to the hippocampal and medial frontal regions for memory and face recognition [52,75]. Limbic responses are known to be greater during explicit (emotion discrimination) rather than implicit (e.g. age discrimination) facial emotion processing [76]. Moreover, since the amygdala plays such an important role in emotional responses and has structural and functional connections to the prefrontal and temporal cortices [77,78], frontal and temporal source activity in our adolescent dipole models possibly reflected underlying amygdala activity triggered by emotional facial expressions.

Finally, we suspect that parietal source activity in young adolescents at roughly 280 ms post-stimulus could represent an engagement of the parietal somatosensory cortices when participants mentally simulated the perceived facial expression in order to achieve an emotional response for a better understanding of the emotion [5,72,79]. It has been shown that a single presentation of a face displaying an emotion for 500 ms is sufficient to elicit an emotional reaction in the observer [79]. We speculate that expressions of happiness, sadness, and fear for example, are likely to have evoked the corresponding emotions, more so in the younger group than in the older adolescents, while angry faces may have triggered feelings of fear and disgust [79].

## Limitations

Although the small sample size in this study may have limited the power to detect significant effects of emotion and age on scalp ERP components and ERP brain source activity, our findings will form the basis for future developmental ERP studies employing spatiotemporal dipole source analysis on a wider age range of children, adolescents, and adults. In particular, frontal and parietal sources of later ERP components (those after 200–300 ms post-stimulus) deserve to be studied in greater detail, as emotion-specific activities are likely to occur at longer latencies in children and adolescents than in adults. Incorporating local maxima of fMRI activations from the same participants as constraints for ERP dipole sources can further improve localization accuracy and optimize the spatial and temporal resolution these complementary modalities provide [80]. It would also be useful to extend this work to populations with social-emotional deficits or abnormalities in processing of faces and emotions, such as autism, depression, and schizophrenia, in order to examine differences in the engagement of neural systems and distinct perceptual/cognitive styles of facial emotion processing.

## Conclusion

This is a first report of spatiotemporal dipole source analysis on healthy adolescent ERP data tracing the sequence of neural activity within the first 500 ms of categorizing emotion from faces. The similarity in source locations between our adolescent models and previous adult findings [11,19] suggest that cortical networks for face processing are present before 16 years of age [53], but their functional specificity to different emotions are not yet fully mature. Differences in brain sources and activation patterns between young and older adolescents illustrate the continuing development and maturation of distinct neural systems for processing emotional facial expressions during adolescence and possible changes in emotion perception, experience, and reaction with age.

## Competing interests

The authors declare that they have no competing interests.

## Authors' contributions

SEC, GMM and PCWF conceived of, acquired funds for, and coordinated the study. TKWW acquired, processed, and analyzed the data, as well as drafted the manuscript. All authors were involved in the design of the paradigm, interpretation of data, and revision of the manuscript. All authors read and approved the final manuscript.
